# Mode of failure and finite element analysis of custom-made PEEK post–core (milled and pressed)

**DOI:** 10.1007/s10266-025-01084-7

**Published:** 2025-03-15

**Authors:** Nour Abdelmohsen, Marwa Wahsh, Maged Zohdy, Ghada Abdelfattah, Abdulaziz Alhotan, Ashraf Refaie, Christoph Bourauel, Tarek M. Elshazly

**Affiliations:** 1 Department of Fixed Prosthodontics, Faculty of Dentistry, University of Ain Shams, Cairo, Egypt; 2 Dental School, Oral Technology, University Hospital Bonn, Welschnonnenstr. 17, 53111 Bonn, Germany; 3 Department of Fixed Prosthodontics, Faculty of Dentistry, Galala University, Suez, Egypt; 4 Department of Dental Health, College of Applied Medical Sciences, King Saud University, Riyadh, Saudi Arabia; 5 Department of Fixed Prosthodontics, Faculty of Dentistry, Fayoum University, Faiyum, Egypt

**Keywords:** Restorative dentistry, Endodontics, CAD/CAM, FEM, Stress analysis, PEEK

## Abstract

To compare experimentally the failure modes of endodontically treated teeth restored with custom-made polyetheretherketone (PEEK) post–core (milled and pressed) to those restored with the standard technique of customized fiberglass posts with composite cores, and to analyze numerically stress distribution patterns in each system using the finite element analysis (FEA). Experimentally, 21 mandibular premolars were endodontically treated, prepared for post-restoration, mounted in resin mold, decapitated, and assigned to three groups: M (milled PEEK), P (pressed PEEK), and C (control group; ready-made fiberglass posts customized with resin composite). All post–core restorations were cemented and subjected to thermocycling. Failure modes were visually evaluated after fracture by means of a universal testing machine. Numerically, stress analysis was assessed using FEA, based on digital models designed to replicate the experimental setup. Milled PEEK posts showed a uniformly favorable failure mode across all specimens (100%), whereas pressed PEEK and customized fiberglass posts displayed more variable failure modes, yet with a higher proportion of favorable outcomes. Numerically, the highest VME stress values were in the cervical half of the post area (up to 62.7 MPa), with minimal differences between milled and pressed PEEK posts. PEEK posts had greater stress concentrations in the cervical area of the post area, while fiberglass posts showed slightly higher stress in the middle and apical areas. Custom-made milled and pressed PEEK posts are effective for restoring endodontically treated teeth, with milled PEEK posts showing the most favorable failure mode and stress distribution pattern.

## Introduction

The selection of treatment options for endodontically treated teeth requires careful assessment and precision [1, 2]. Endodontic treatment affects the mechanical and physical properties of the tooth making it more fragile and fracture-prone [3–5]. When the remaining coronal tooth structure is insufficient to retain the core following decay removal and access preparation, the use of a post is indicated in such cases [6–8]. A prefabricated post is the primary treatment option for cases with a regular circular canal morphology due to its availability and ease of application [9].

In cases of non-circular, wide, or irregularly shaped canals, a prefabricated post cannot be used unless it is customized to improve its fit within the canal. Alternatively, a custom-made post can be fabricated to conform precisely to the canal’s anatomy [10–12]. For many years, gold custom-made post–core was considered the treatment of choice for such cases [13–15]. However, its use is restricted by several factors, including their poor esthetics when combined with highly translucent ceramic crowns [15] as well as the relatively high cost of gold [14]. More importantly, the substantial difference in the elastic modulus between the post and root dentin creates stress concentrations within the root, increasing the likelihood of vertical root fractures and irreversible damage, which may ultimately necessitate tooth extraction [16–18].

Furthermore, prefabricated fiberglass posts, which can be customized with resin composite, are increasingly utilized. Fiberglass posts have a lower elastic modulus compared to metal but still provide adequate strength, facilitating favorable stress distribution within the root and typically resulting in a more repairable horizontal fracture mode if the fracture occurs [17, 18]. However, their elastic modulus remains significantly higher than that of dentin [20]. In addition, the use of customization composites with different properties, along with the interface between these materials, can introduce new sites for stress concentrations [10].

Recent advancements have introduced alternative biocompatible, tooth-colored materials designed to overcome the previously discussed drawbacks [21, 22]. Among these, high-performance polymers, with an elastic modulus lower than fiberglass and very close to dentin, have emerged as alternatives for intra-radicular post–core materials [23]. These high-performance polymers are noted for their adequate fracture resistance and enhanced shock absorption [23, 24]. Polyetheretherketone (PEEK) is a member of the high-performance polymer family, characterized by its white color when reinforced by ceramic particles and high thermal stability up to 335.8 °C. The elastic modulus of PEEK and tensile properties closely match those of human bone and dentin [23, 24]. It has been increasingly used in dentistry to replace traditional materials such as ceramics and metals in the fabrication of removable and fixed prostheses [24–27]. PEEK can be processed through milling or pressing techniques [28].

Finite element analysis (FEA) is a crucial research tool for biomechanical studies in biological research [29, 30]. This engineering technique is used for structural analysis of bodies with irregular geometries and heterogeneous material properties. It involves discretizing the structure into a finite number of elements connected by nodes. By applying an appropriate mesh and selecting a suitable mathematical model for each element, FEA enables the computation of reactions and interactions within the structure [31, 32]. Compared to physical testing, FEA offers a more economical, repeatable, and time-efficient approach without altering the physical properties of the materials used [32–35]. This makes it highly effective for accurately assessing stress distribution in post–core systems [20].

While numerous articles [19–22] discuss the finite element biomechanical analysis of dental post–core systems, relatively few studies specifically address PEEK post systems [36]. The literature provides limited data on the effectiveness of milled and pressed PEEK posts compared to customized fiberglass posts in restoring endodontically treated teeth without increasing the risk of non-repairable root fractures. Therefore, this study employs a combination of experimental and numerical approaches to examine the failure modes and stress distribution in endodontically treated teeth restored with custom-made PEEK post–core systems (both milled and pressed), introducing the novel comparison to customized fiberglass posts with composite cores. Experimental fracture testing was conducted to evaluate failure modes, while finite element analysis (FEA) was employed to analyze stress distribution patterns that cannot be fully captured through experimental methods alone. The first null hypothesis assumes no differences in failure modes among the three post–core systems, and the second null hypothesis suggests no differences in stress distribution patterns.

## Materials and methods

### Experimental part (fracture test)

This study was approved by the Ethics in Research Committee at the Faculty of Dentistry, Ain Shams University, Egypt (approval number FDASU-ReclE121904). A total of 21 human mandibular premolar teeth extracted for orthodontic or periodontal reasons were collected for this study. All teeth showed fully developed apices and had no caries, cracks, restorations, erosion, abrasion, or fractures. They had similar root form and root canal shape, averaging cervico-occlusal length of the crown about 8.0 ± 0.5 mm and root length of about 14.5 ± 0.5 mm. Teeth were carefully cleaned and stored in distilled water at room temperature until used.

Using parallelometer dental surveyor, each root was embedded in Acrostone acrylic resin (Acrostone dental and medical supplies, Heliopolis, Egypt) perpendicular to the floor with zero angle tilt up to 2.0 mm short of the cemento-enamel junction (CEJ), using a circular polyvinyl chloride (PVC) cylinder (25.0 mm in diameter/ 20.0 mm high). The set (tooth, cylinder, and resin) remained stable for 72 h to ensure full setting of the resin. Decapitation of the crowns was done 2.0 mm above the level of CEJ with a low-speed diamond disc operating at 25,000 rpm (Dental Fix, Mississauga, Canada) [37].

Root canals were prepared using ProTaper gold rotary files (Dentsply international Inc., Johnson, Tennessee, USA) 1.0 mm short of the apex. Afterward, the canals were irrigated intensely using 10.0% sodium hypochlorite solution Clorel (Alexandria detergents and chemicals Co., Alexandria, Egypt) and dried with absorbent paper points ProTaper gold paper points (Dentsply international Inc., Johnson, Tennessee, USA). Using the cold lateral compaction technique, filling root canals were carried out with ProTaper gold conform Fit gutta-percha (Dentsply international Inc., Johnson, Tennessee, USA) and resin-based sealer (ADSEAL; META BIOMED, Chungcheongbuk-do, Korea) [14].

Post space drilling was done using size #2 and #3 LARGO pesso reamer (Dentsply Maillefer, Ballaigues, Switzerland) to remove 8.0 mm of the gutta-percha. Without using coolant, this process was implemented because the heat generated by the drill enhanced gutta-percha removal. To give the drilling space a uniform conformity, the Rely-X fiber post blue drill (3M ESPE, Seefeld, Germany) was used to finish the preparation of the root canals (8.0 mm in length and 0.9 mm in width apically, 5.72° (10.0%) taper). After the preparation of each root, the corresponding teeth were allocated randomly to three groups (*n* = 7 per group) according to the type of material and fabrication technique used to generate the post–core: group M; milled PEEK, group P; pressed PEEK, group C; fiberglass posts customized with resin composite (control). For the purpose of standardization, all the steps were carried out by the same well-trained operator.

The teeth of groups M and P were scanned using CEREC Omnicam intraoral scanner (CEREC premium SW4.4; Dentsply Sirona, Bensheim, Germany), with a total scanning depth of 10.0 mm [38]. Using Sirona connect application, the produced scans were exported to inLab software 15.1 (Dentsply Sirona, Bensheim, Germany), where the post–core systems were designed. A cement space of 85.0 μm was selected. The margins of the core were designed 1.0 mm away from the external enamel margin of the tooth with a height of 4.0 mm.

**Fig. 1:**
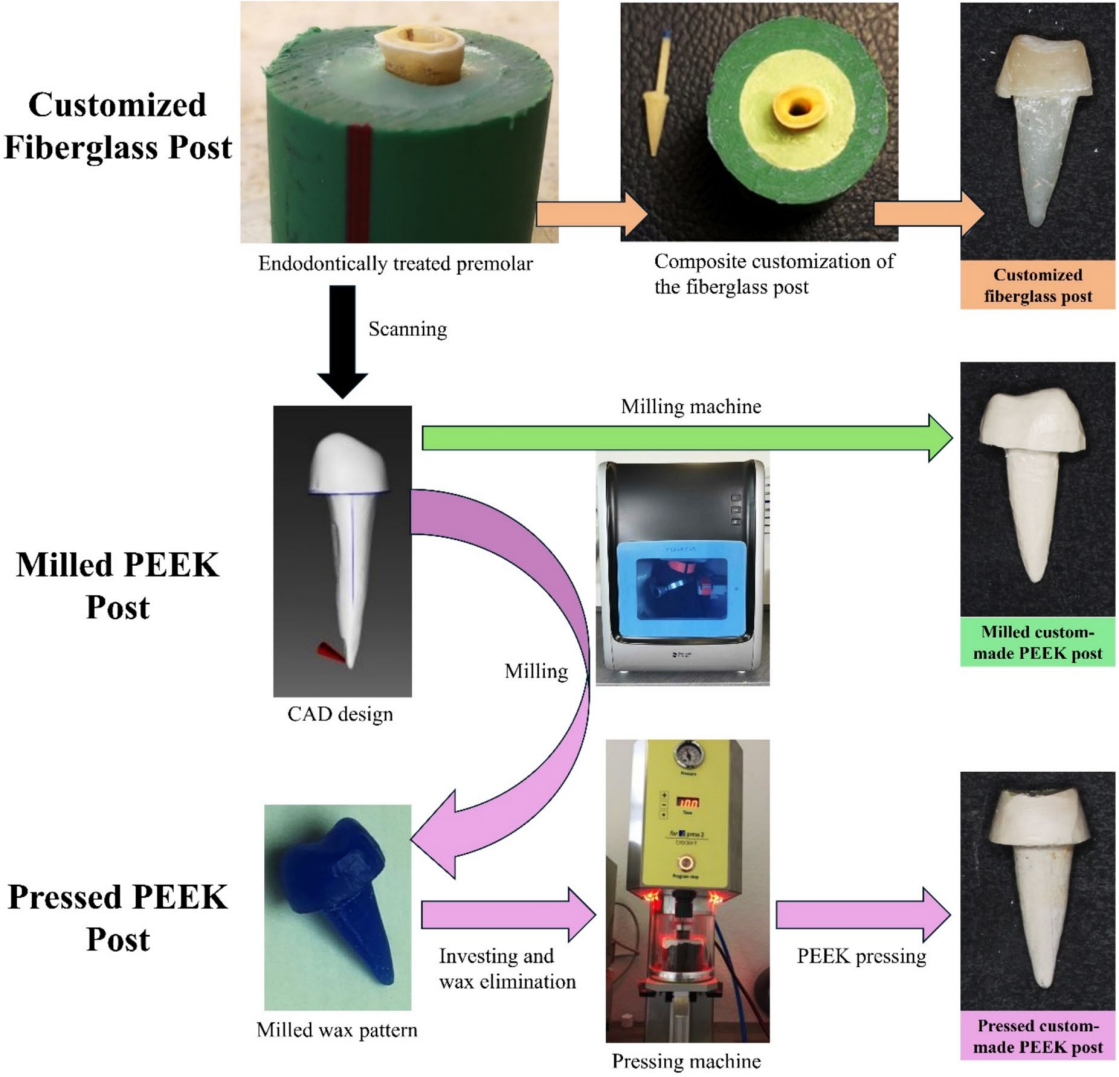
Schematic workflow diagram illustrating steps followed for post–core production of different systems14

For the M group, the designs were milled from BioHPP PEEK blanks (Bredent GmbH & Co., Senden, Germany) into final restorations using the inLab MCX5 milling machine (Dentsply Sirona, Bensheim, Germany). In the P group, the computer-aided design (CAD) files were milled into wax patterns (CopraWax, Whitepeaks Dental Solutions GmbH, Hamminkeln, Germany) using the inLab MCX5 milling machine. The wax patterns were then invested using a special phosphate-bonded investment material (Gilvest powder and liquid; SRL Dental GmbH, Ludwigshafen, Germany). Preheating for melting the wax and managing the expansion of the investment material was performed in the laboratory’s pre-heating oven at 800.0 °C. After complete melting, the temperature was maintained at 400.0 °C for 20 min. The pressing procedure for BioHPP PEEK granules (Bredent GmbH & Co., Senden, Germany) was carried out fully automatically using the For2press system (For2press system; Bredent GmbH & Co., Senden, Germany) and completed within 35 min, after which the restorations were devested (Fig. 1).

For the C group, seven blue-coded Rely-X fiber posts (3M ESPE, Seefeld, Germany) measuring 1.9 mm at the coronal end and 0.9 mm at the apical end were used. The surfaces of the posts were thoroughly cleaned by immersion in 70.0% alcohol for 1 min, dried using sterile gauze, and then coated with a layer of silane coupling agent (Calibra; Dentsply Maillefer, Petrópolis, RJ, Brazil). Customization was carried out using Amaris resin composite (VOCO, Cuxhaven, Germany). The resin composite was applied in layers to the post surface, and the post/composite assembly was subsequently inserted into the root canal, which had been previously coated with a water-soluble gel (KY Gel; Johnson & Johnson, Sao Paulo, Brazil) to serve as a separating medium. After customization, the fiber post head was trimmed 4.0 mm above the coronal end of the specimen to facilitate core fabrication. For standard composite core production, a mold was created with transparent CharmFlex silicone bite registration material (Dentkist Inc., Gyeonggi-do, Korea). The mold was formed over one of the milled PEEK cores [39] from a previous M group. A Universal Tofflemire Matrix Retainer and its band (House Brand Dentistry, Concord, Canada) were placed around the coronal part of the prepared tooth and core. The bite registration material was injected with a small intraoral tip around the core up to the border of the band and left to set completely. For core production, the tofflemire band and holder were used to encircle the teeth to ensure standardized positioning of the mold, and the mold was then positioned over the trimmed fiber post within the confines of the band and filled with a core build-up material (Dentocore; Itena, Villepinte, France), followed by light curing of the core, using LED.H device (Woodpecker; Muenster, Germany) for 40 s according to the manufacturer’s instructions (Fig. 1).

It is known that PEEK has an inert surface, making the bonding procedure a challenging step [25, 40]. Hence, for M and P groups, the cementation process for BioHPP posts followed the manufacturer’s guidelines. Air abrasion was performed first, with the posts being blasted with 110.0 μm aluminum oxide at 2.5 bar pressure from a distance of 3.0 cm. The posts were then coated with a Visiolink primer layer (Bredent GmbH & Co., Senden, Germany) and left for a few minutes to allow solvent evaporation, and light-cured for 90 s. For group C, before cementation, the root canals were rinsed with 2.0 mL of distilled water to remove any residues of the water-soluble gel. They were then dried with a triple syringe using oil-free air, and absorbent paper points were used to ensure complete dryness. The posts were sandblasted with aluminum oxide for 5 s at a distance of 30.0 mm and 2.0 bar pressure and then cleaned in an ultrasonic machine.

All post–core systems were cemented using self-etching resin cement G-CEM dual cure capsules (GC Asia, Changi, Singapore). Initial Tac curing for 5 s was carried out using LED.H light curing device, excess cement was then removed, final curing was carried out for additional 20 s on each surface. Afterward, specimens underwent thermocycling, which involved immersion in two water tanks with temperatures of 5.0 °C (cold) and 55.0 °C (warm), alternating every 15 s, with a 5-s water drip in between, for a total of 5,000 cycles. This procedure simulates approximately 6 months of clinical use [40–43].

A universal testing machine (WTS; Jinan, Shandong, China) with a 1.0 mm diameter blunt-end tip was used to apply a progressively increasing load along the long axis of the specimens at a crosshead speed of 1.0 mm/min until fracture occurred. Through magnifying loops assessment (Carl Zeiss, Oberkochen, Germany) and periapical radiograph evaluation, the mode of failure of each specimen was assessed and categorized according to Falcao et al. [44] classification (Table 1), as either favorable (restorable) or non-favorable (catastrophic), based on the restorability of the tooth [45, 46]. Horizontal or oblique fractures at the crestal bone level (Types 0, 1, 2, 3, and 4) were considered favorable, while more apical root fractures (Type 5) were deemed catastrophic and non-restorable [45, 46]**.**

**Table 1:** Classification of tooth failure mode after load application until fracture [44]

Failure mode	Description
Type 0	Absence of visual damage (no failure)
Type 1	Fracture, crack, or chip up to 50% of the coronal portion of the core or crown, without involvement of the post portion (favorable)
Type 2	Fracture, crack, or chip of more than 50% of the coronal portion of the core or crown, without involvement of the post portion (favorable)
Type 3	Fracture or crack of the coronal portion of the core or crown, with the involvement of the post portion, but without involvement of the root dentin (favorable)
Type 4	Any type of fracture involving the root dentin at the cervical third (above bone level) (favorable)
Type 5	Any type of fracture involving the root dentin at the middle or apical thirds (non-favorable; catastrophic)

### Numerical part (finite element analysis)

A sample from the M group, along with its designed restoration, was used to create a 3D model of the mandibular premolar based on data obtained from a computed tomography (CT) scan performed with the I-CAT Next Generation scanner (Imaging Sciences International, Hatfield, PA, USA). The scan was conducted at a tube voltage of 120.0 kVp, a tube current of 5.0 mA, with a voxel size of 0.2 mm and a field of view measuring 16.0 × 6.0 cm, taking 26.9 s to complete. The resulting digital Imaging and Communications in Medicine (DICOM) file was imported into SOLIDWORKS 18 (Dassault Systèmes, Waltham, Massachusetts, USA), a 3D mechanical CAD and simulation software, to generate the finite element model of the premolar. Using the software’s design tools, a 0.2 mm periodontal ligament (PDL) was created around the root, surrounded by a block of supporting alveolar bone measuring 12.0 × 9.0 × 18.0 mm [3], positioned at an intact bone level 2.0 mm below the cemento-enamel junction (CEJ).

**Fig. 2:**
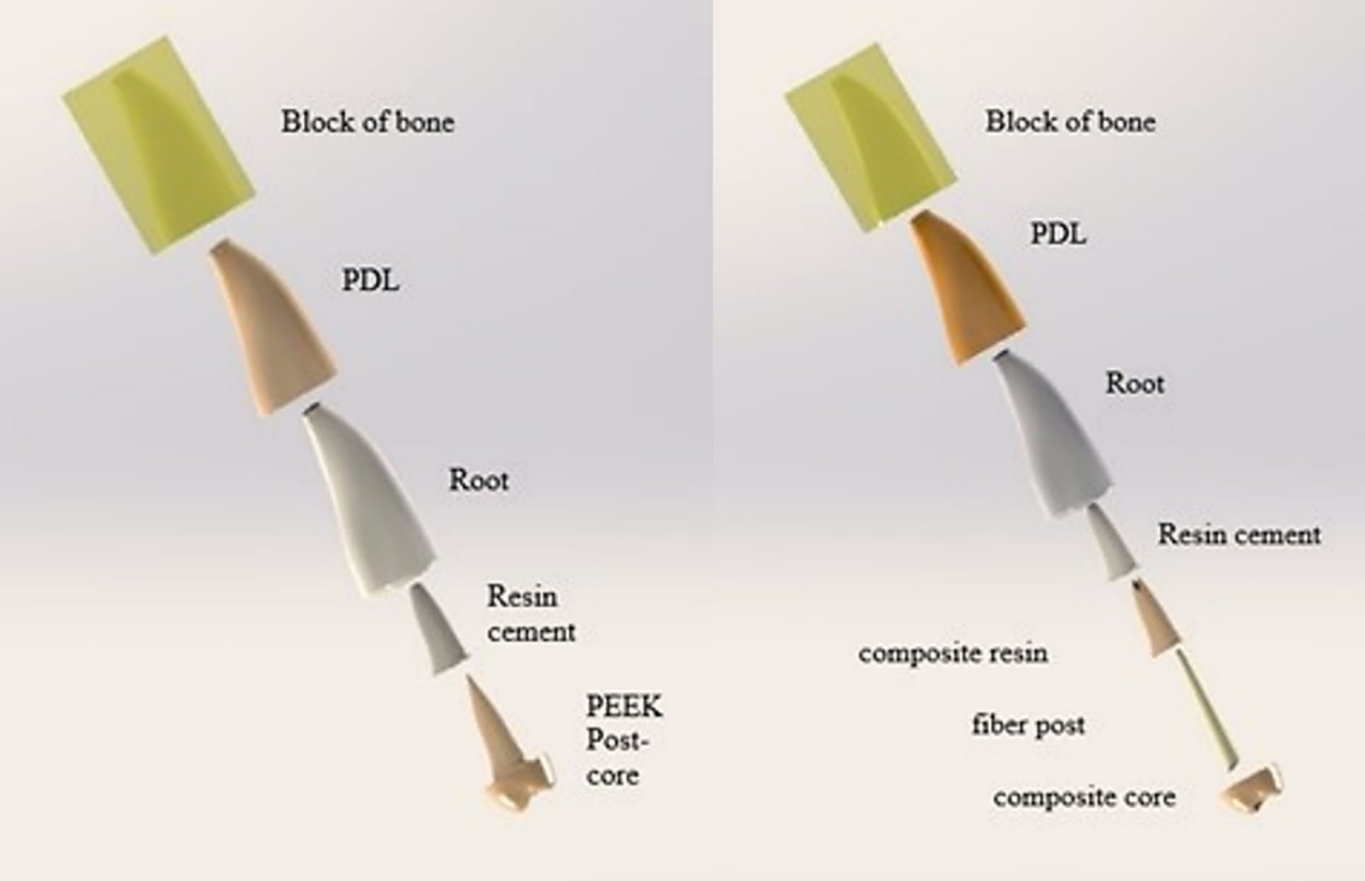
Components of finite element models; custom-made milled and pressed Polyetheretherketone (PEEK) posts (left), customized fiberglass post (right)17

**Fig. 3:**
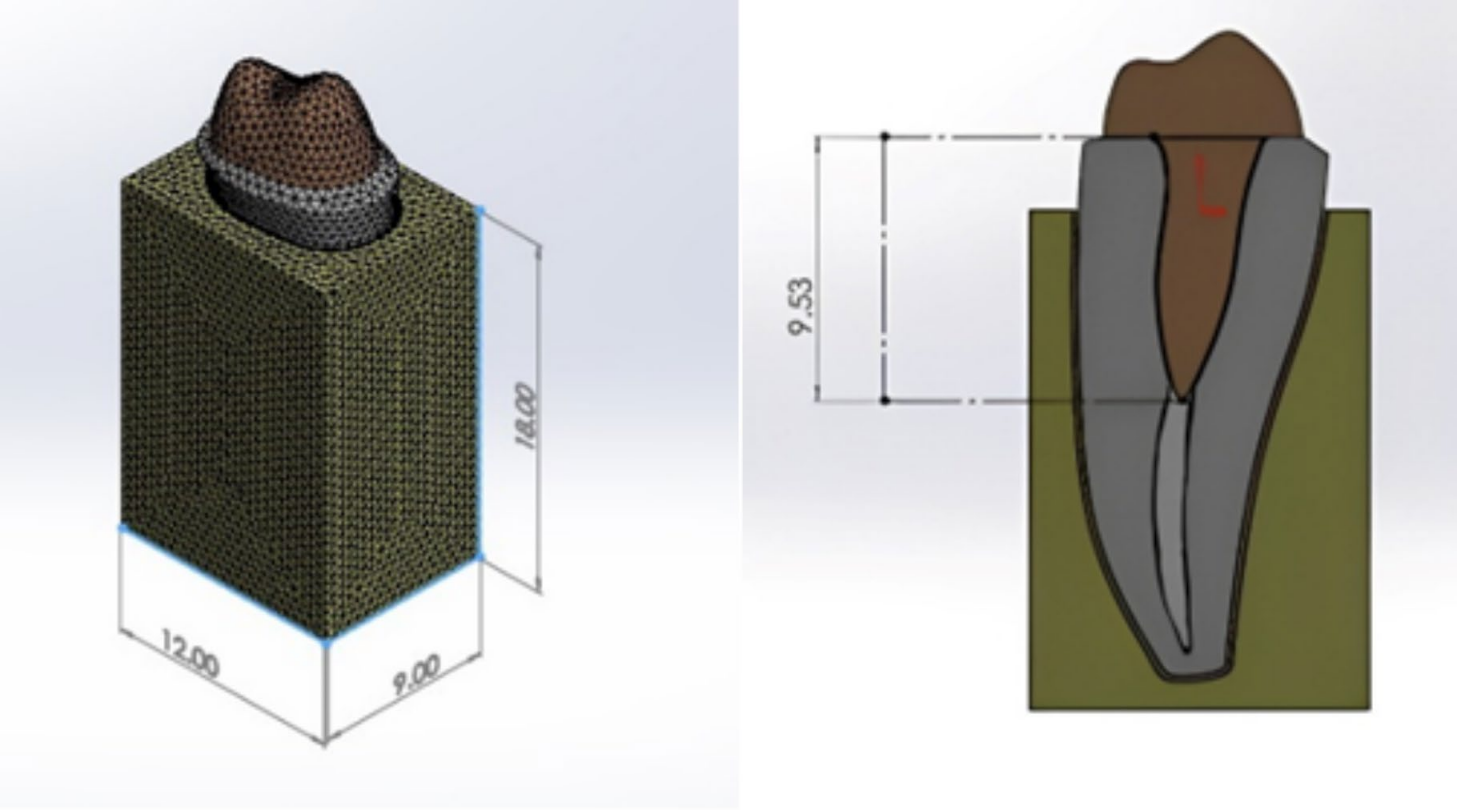
The generated 3D mesh of finite element model using SOLIDWORKS, showing dimensions of investing block of bone in mm (left) and a cross-sectional view showing the post dimensions in mm (right)18

The corresponding post–core designed restoration was exported as a Standard Tessellation Language (STL) file to SOLIDWORKS and adjusted over the tooth to form the finite element model for both milled and pressed PEEK custom-made post–core systems. For the customized fiberglass post, a tapered cone with the same dimensions as the blue Rely-X fiberglass post (8.0 mm in length, 0.9 mm in width apically, with a taper of 5.72° (10.0%)) was drawn centrally within the design of the PEEK post–core, representing the fiberglass post. The space between the post and root dentin was assumed to be filled with customization composite, the coronal part of the restoration was assumed to be core composite, and the cement gap between the post and the root was assumed to be the cement layer in both models (Figs. 2 and 3).

Accordingly, in this study, three different post–core systems were compared using two types of geometry models, as shown in Fig. 2. One of the geometry models was a one-piece post–core model to exhibit a PEEK post–core system fabricated by two techniques: pressing and milling. The other geometry model was a fiberglass post, a customization composite, and a separate core model to exhibit a customized fiberglass post system. The shape of all post–core systems in this study was considered the same, to assess only the effect of the post–core materials, excluding the effect of dimensions.

The length of the designed post was approximately half the root length, the other half of the root was filled with gutta-percha (GP) cone. Finally, the elements of the constructed geometry model consist of an alveolar bone, periodontal ligament (PDL), tooth root, GP cone, resin cement, and one-piece post–core (or composite-customized fiberglass post and resin core). The 3D meshes were generated using a blended curvature-based mesher with four Jacobian points and an element edge length of 0.1032 to 0.516 mm on average. Thus, the meshing procedure generated around 230,000 nodes and 150,000 hexahedral elements, as shown in Fig. 3.

In this model, the mechanical properties of all materials were assumed to be homogeneous, isotropic, and linear elastic for simplicity, except for the fiberglass post. The fiberglass post was considered orthotropic due to its varying mechanical properties along the fiber direction (x-direction) compared to the two perpendicular directions (y- and z-directions). The applied values were derived from previous studies, as detailed in Table 2 [7, 47]. All assemblies were assumed to be fully bonded, and the bottom of the alveolar bone was fixed to prevent any rigid dynamic motions. A vertical force of 300.0 N, representing the average occlusal load [49], was applied to a group of ten central nodes on the core’s outer surface, perpendicular to the occlusal plane and parallel to the tooth’s longitudinal axis. To identify regions of highest stress intensity, equivalent von Mises (VME) stresses were calculated for each component of the numerical models, and the resulting stress distributions were analyzed and plotted.

**Table 2:** The mechanical material properties of the finite element models

Material	Elastic modulus (GPa)	Poisson's ratio	Reference
Cancellous bone	1.37	0.30	47
Periodontal ligament (PDL)	0.05	0.45	7
Dentin	18.60	0.31	47
Gutta-percha	0.14	0.45	7
Resin cement G-CEM	7.20	0.30	Manufacturer
Fiberglass post	E_x_ = 37.00	ν_yz_ = 0.34	7, 20
	E_y_ = 9.50	ν_xz_ = 0.27	
	E_z_ = 9.50	ν_xy_ = 0.27	
Resin composite	12.00	0.33	7
Milled BioHPP PEEK	4.55	0.37	Manufacturer
Pressed BioHPP PEEK	4.00	0.37	Manufacturer

### Statistical analysis

A power analysis was performed based on the results of Maroulakos et al. 41. The calculated sample size was 21 specimens in total, corresponding to 7 specimens per group. The effect size (f) was determined to be 0.81. Using an alpha (α) level of 0.05 and a beta (β) level of 0.20, corresponding to a statistical power of 80.0%, the total sample size was confirmed to be 21. The sample size calculation was conducted using G*Power software version 3.1.9.4 (IBM Corporation, New York, NY, USA) 50. Categorical data were presented as frequency and percentage values and were analyzed using Fisher’s exact test. The significance level was set at *p* < 0.05. Statistical analysis was performed using SPSS Statistics Version 26.0 for Windows (IBM Corporation, New York, NY, USA).

## Results

### Experimental part (mode of failure)

**Fig. 4:**
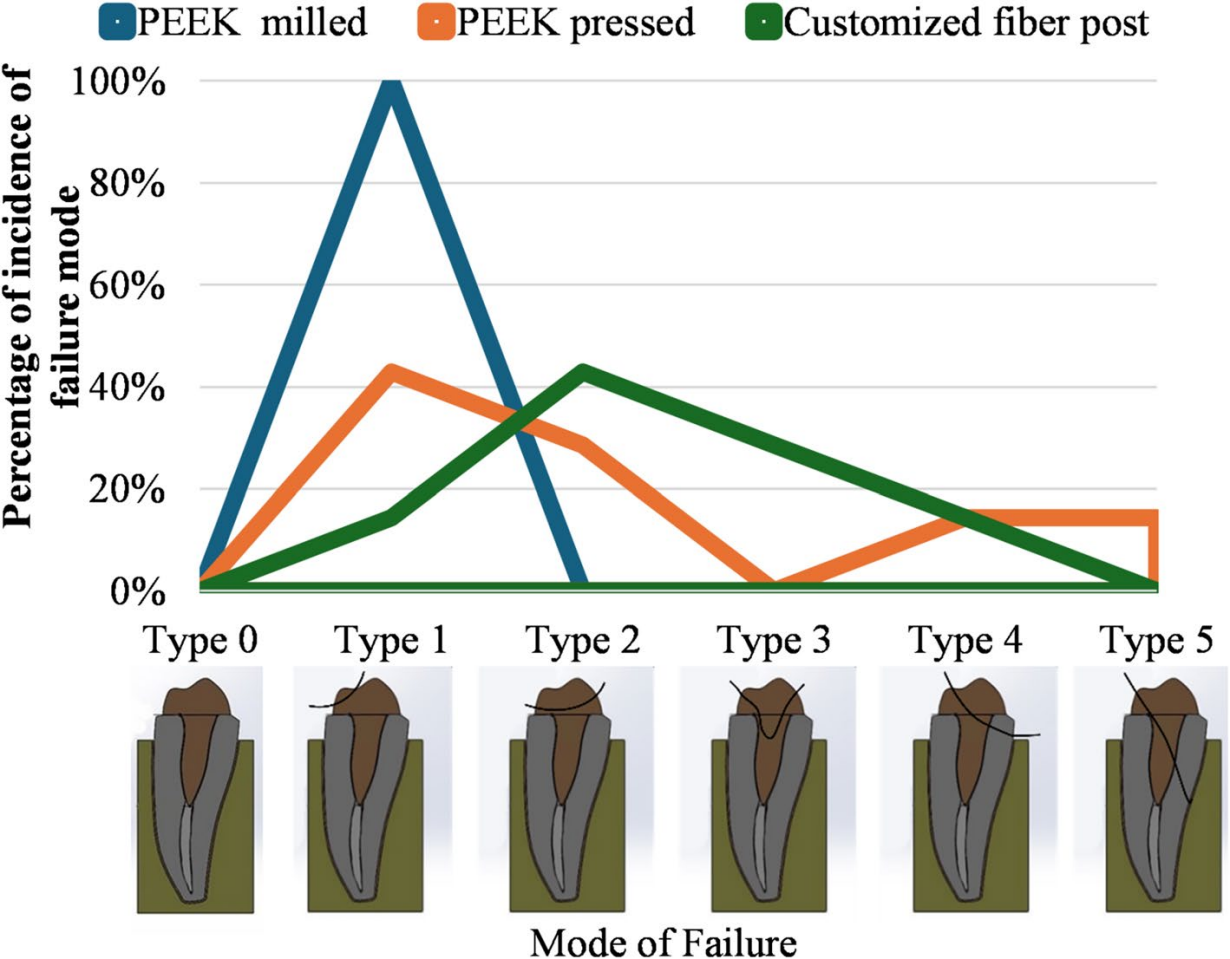
Graph showing the percentage of incidence of failure mode in different groups, Type 0; absence of visible damage, Type 1; fracture of core less than 50.0%, Type 2; fracture of core more than 50.0%, Type 3; fracture of core and part of post area without tooth involvement, Type 4; fracture involving cervical 1/3 of the root, Type 5; fracture involving middle and apical 2/3 of the root

The percentage (%) of incidence of failure modes across different groups is shown in Table 3 and Fig. 4. Milled PEEK displayed consistent results, with all samples (7 samples, 100%) showing the most favorable failure mode, classified as Type 1. In contrast, pressed PEEK and customized fiber posts exhibited varied failure modes, mostly favorable but with a lower percentage than milled PEEK. For pressed PEEK, 3 samples (42.9%) demonstrated Type 1 failure, 2 samples (28.6%) showed Type 2 failure, while one sample (14.3%) showed Type 4, and another (14.3%) showed Type 5 failure. For customized fiber posts, 3 samples (42.9%) exhibited Type 2 failure, 2 samples (28.6%) showed Type 3 failure, while one sample (14.3%) showed Type 1 failure, and another (14.3%) exhibited Type 4 failure.

**Table 3:** Frequencies (n) and percentages (%) of mode of failure in different groups

Failure mode		PEEK milled	PEEK pressed	Customized fiber post	*p* value
Type 0	n	0^a^	0^a^	0^a^	0.025*
	%	0%	0%	0%	
Type 1	n	7^b^	3^ab^	1^a^	
	%	100.0%	42.9%	14.3%	
Type 2	n	0^a^	2^a^	3^a^	
	%	0.0%	28.6%	42.9%	
Type 3	n	0^a^	0^a^	2^a^	
	%	0.0%	0.0%	28.6%	
Type 4	n	0^a^	1^a^	1^a^	
	%	0.0%	14.3%	14.3%	
Type 5	n	0^a^	1^a^	0^a^	
	%	0.0%	14.3%	0.0%

Different superscript letters indicate a statistically significant within the same horizontal row*; significant (*p* ≤ 0.05). *ns* non-significant (*p* > 0.05).

### Numerical part (finite element analysis)

**Fig. 5:**
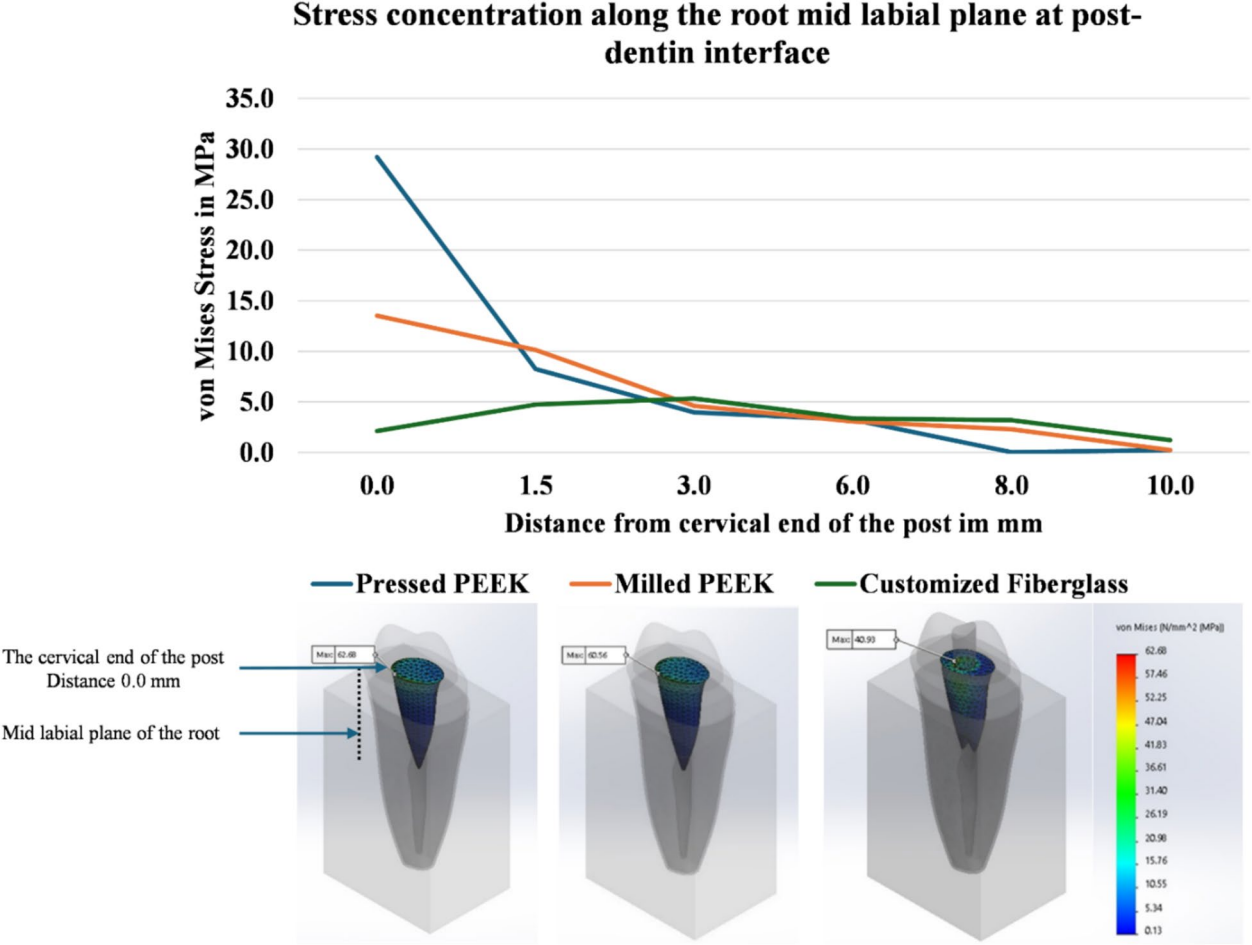
A graph presents the equivalent von Mises (VME) stresses along the mid-labial plane of the post-dentin interface, plotted against the distances measured from the cervical region to the apex of each post. Arrows in the models mark the nodes with the highest stress values in the post region; milled PEEK (left) and pressed PEEK (middle) showing the highest stress point cervically at post–dentin interface, while the customized fiberglass post (right) showing the highest stress point cervically at fiber post–customization composite interface.25

In both PEEK and fiberglass post models, the highest VME stress values were in the cervical half of the post area (Fig. 5). Stresses were determined along the root mid-labial plane of the post–dentin interface. For the milled and pressed PEEK posts, the post–dentin interface was the primary area of high-stress concentration, whereas in the customized fiberglass post, the highest stress concentration occurred along the interface between the fiberglass post and the customization composite (Fig. 5). Minimal differences were observed between the milled and pressed PEEK posts throughout the post length, while both demonstrated higher stress concentrations in the cervical one-third compared to the customized fiberglass post. Conversely, the customized fiberglass post showed slightly higher stress values in the middle and apical thirds of the post (Fig. 5).

## Discussion

Root fracture is a highly undesirable outcome for an endodontically treated tooth restored with a post–core. Previous studies 15, 18 have indicated that one of the primary causes of root fractures in post-restored teeth is the concentration of stress around the post-apex. Therefore, analyzing the pattern of stress dissipation and the capacity of restorative materials to prevent stress concentration can offer valuable insights into the causes of failure and help predict potential points of fracture. In this study, the high-performance polymer PEEK, in both milled and pressed forms, was evaluated as an intra-radicular post–core material and compared with conventional commonly used customized fiberglass post–core materials.

In this study, natural teeth were used to closely replicate the actual canal space and to enable proper endodontic treatment. Amarnath et al. 51 reported that the type and length of the post within the root are critical factors in the success of post–core-restored endodontically treated teeth. Hence, twenty-one mandibular premolars with almost similar dimensions were selected to minimize the effect of variations in post length and width on the tested parameters. In addition, given that direct digital acquisition systems are faster, less invasive, and more accurate, digital scanning was employed in this study instead of traditional impression techniques 52, 53. For the pressed PEEK group, computer-aided design/computer-aided manufacturing (CAD/CAM) technology was used to generate wax patterns, since the subtractive waxing technique provided superior internal and marginal fit accuracy compared to conventional wax pattern production methods 54.

In both experimental and numerical analyses, a restorative crown was deliberately omitted to eliminate its potential protective effect. This exclusion ensured that the failure and stress analyses focused solely on the tested post materials, without interference from the load-distributing properties of a crown, thereby providing a more accurate assessment of the biomechanical behavior of the posts. Especially given that the full-coverage crowns have been reported to enhance the fracture resistance of endodontically treated teeth restored with fiberglass posts 51. As outlined by Falcao et al. 44 and summarized in Table 1, fractures of endodontically treated teeth are classified into five types based on the tooth’s restorability after fracture. Types 1, 2, 3, and 4 are favorable, meaning the tooth can be restored, while Type 5 is unfavorable, requiring extraction. Within the favorable categories, Types 1 and 2 generally do not require post retrieval, making them easier to manage with simple clinical repairs of the fractured core. On the other hand, Types 3 and 4, which involve post fractures, often necessitate post retrieval and replacement, requiring more advanced clinical procedures.

A universal testing machine was utilized to assess the mode of failure. A vertical load was applied because it better evaluates the cohesive properties of the tested materials and distributes stress more evenly between the dental tissues and restorative materials, thereby simulating physiological occlusion 55. The first null hypothesis was rejected due to a significant difference in the distribution of failure modes among the groups. Milled and pressed PEEK exhibited favorable failure modes due to the material’s homogeneity and the reduced number of interfaces, unlike customized fiberglass posts, which showed less favorable failure patterns due to multiple stress concentration areas (interfaces) that served as initial failure sites 56. Milled PEEK demonstrated a more consistent and favorable mode of failure compared to pressed PEEK. This consistency can be attributed to milling being a more standardized process, since milling blanks are pre-pressed industrially, which enhances their mechanical properties and reduces the risk of porosities in restorations under optimal manufacturing conditions [55]. On the contrary, the mechanical properties of pressed restorations are more operator-dependent, with factors such as the pre-heating process, vacuum pressing equipment, and other variables influencing the overall quality of the final product [55].

The second null hypothesis was rejected as the PEEK and fiberglass post models exhibited dissimilar stress distribution patterns and different values of maximum VME stress within the post area. The stress distribution was analyzed using FEA along the post length in all models in terms of equivalent von Mises stress (VME). Although VME was initially developed to evaluate the behavior of ductile metallic materials, it is widely applied in biomechanics to identify areas of high concentration of stresses, where the specific direction of stress (tensile, compressive, or shear) is not critical.

In the PEEK models, both milled and pressed PEEK post–core systems demonstrated favorable stress patterns, with stress concentration primarily observed in the cervical one-third. This stress distribution pattern reduces the risk of non-repairable failures, such as vertical root fractures, thereby minimizing the likelihood of tooth extraction. These findings align with the failure mode results for the milled PEEK group, which consistently exhibited favorable failure patterns. However, the numerical results for the pressed PEEK group showed some discrepancies with the experimental outcomes. This divergence may be attributed to the numerical analysis assuming homogeneous and isotropic material properties, whereas the pressing process introduces slight variations in consistency and quality compared to the milling process.

In the customized fiberglass post model, higher stress values were observed in the apical two-thirds of the post, increasing the risk of severe root failure [56] , which may explain the occurrence of Type 3 and 4 failure modes in the C group, in alignment with a study by Lima et al. 22. This can be referred to the higher elastic modulus of fiberglass post compared to root dentin and PEEK. The customized fiberglass post also has the disadvantage of multiple interfaces, particularly between the fiberglass post and the customization composite, as well as between the composite and root dentin. These multiple interfaces create multiple sites for stress concentration 56. The FE model showed stress concentration at the interface between the fiberglass post and the customization composite, mostly due to inadequate adhesion between these components, which increases the risk of failure at this location.

This study has several limitations. First, the experimental design did not fully replicate clinical conditions due to the absence of certain factors, such as cyclic loading to simulate the chewing process and the periodontal ligament (PDL), which serves as a cushion and stress absorber. Incorporating PDL would indeed have enhanced the anatomical relevance of the designed model. However, achieving a standardized 0.2 mm thickness of polyvinylsiloxane (PVS) is a challenging step raising the potential for added errors in the testing procedure. Nonetheless, all samples were tested under identical conditions, ensuring the validity of the comparisons. Other intraoral conditions, such as pH fluctuations and the presence of saliva factors, were also not simulated. Second, numerically, the materials were modeled as homogeneous, isotropic, and linearly elastic to simplify the testing process and analysis of results. Furthermore, the lack of coronal coverage design resulted in higher stress values than would not be expected with coronal coverage. Hence, further laboratory and clinical studies, including the construction of various types of coronal coverage and cyclic loading, are needed to validate these findings.

## Conclusion

Based on the results and limitations of this study, the following conclusions can be drawn:

1. Custom-made milled and pressed PEEK posts are suitable for use as intracanal post materials and can be effectively utilized in the restoration of endodontically treated teeth without posing a risk of non-repairable root failure.
2. The custom-made milled PEEK post–core demonstrated the most favorable and consistent failure mode and stress distribution pattern.

## Clinical implications

The current study offers evidence-based data, both experimental and numerical, on the efficacy of PEEK post-core systems. It recommends using milled PEEK for restoring teeth having wide and irregularly shaped endodontically treated canals, presenting it as an excellent alternative to the conventional customized fiberglass post technique.

## Data Availability

Available on request.
